# Supplementary material for: Are carbon emissions trading and green financial instruments synergistic? -Comprehensive quantitative research based on content analysis
Source: PLoS One. 2024 Mar 7;19(3):e0298601. doi: 10.1371/journal.pone.0298601 (PMC10919601; doi:10.1371/journal.pone.0298601)
Supplement: S1 Data — (ZIP) [file pone.0298601.s001.zip › Data/地级市Z_F工作报告.zip!/░▓╗╒/╗╞╔╜╩╨/└┤╫╘╣1⁄2╓┌║┼í╛┬φ┐╦╩2╛▌═°í┐_2016.docx]

各位代表：

现在，我代表市人民政府，向大会作工作报告，请予审议，并请市政协委员和其他列席人员提出意见。

一、“十二五”时期工作回顾

“十二五”时期是经济进入新常态，各项工作充满挑战的五年；也是全市上下奋力拼搏，经济社会发展取得积极成效的五年。我们深入学习贯彻党的十八大、十八届三中、四中、五中全会和习近平总书记系列重要讲话精神，全面落实党中央、国务院和省委、省政府的决策部署，在市委的坚强领导下，在市人大及其常委会的法律监督、工作监督和市政协的民主监督下，坚持以提高经济发展质量和效益为中心，全力以赴稳增长、促改革、调结构、惠民生、防风险，实现了经济持续健康发展和社会和谐稳定，完成了“十二五”规划确定的目标任务，为“十三五”发展打下了坚实基础。

——过去的五年，是我市综合实力不断增强的五年。始终坚持发展第一要务，主动认识适应引领新常态，紧紧扭住实体经济这个根本，全力推进提质增效、转型升级，经济社会保持健康发展的良好势头。初步测算，2015年地区生产总值530亿元，“十二五”时期年均增长9.4％；人均地区生产总值6205美元，高于全省平均水平313美元；财政收入92.5亿元，五年翻了一番；全社会固定资产投资556.3亿元，年均增长13.6％；社会消费品零售总额218.5亿元，年均增长16.1％；外贸进出口总额6.25亿美元，年均增长13.8%；市内金融机构贷款余额566.3亿元，年均增长15%；住户人均储蓄存款38884元，居全省第4位；城镇常住居民人均可支配收入26250元，年均增长11.6％；农村常住居民人均可支配收入11980元，年均增长13.7%。

——过去的五年，是我市转型升级步伐加快的五年。始终坚持调结构转方式促升级，精准发力、聚焦突破，着力“做精一产、做强二产、做优三产”。大力发展现代农业，特色产业基地240万亩，比“十一五”末增加25万亩；农民专业合作社1639家，是“十一五”末的2.46倍；规模以上农产品加工企业产值190.8亿元，是“十一五”末的3.7倍；农业增加值55.8亿元，年均增长4.1%。全省首家茶产业技术研究院成立，建成90余条茶叶清洁化生产线。黄山区、休宁县分别跻身国家现代农业示范区、全国农业产业化示范基地。新增国家地理标志保护产品8个，总数列全省第一。制定实施《黄山市工业转型升级规划》，规上工业企业达520户，其中产值超亿元企业150户、较“十一五”末增加80户；规上工业增加值127.1亿元，年均增长13.9%；战略性新兴产业产值占规上工业产值比重超过25%。“十二五”时期，全市各类园区累计开发面积40.8平方公里，规上工业总产值占全市比重88.8%，较“十一五”末提高28个百分点；永新股份公司进入中国创新型企业百强。坚持旅游、文化、生态三位一体融合发展，皖南国际文化旅游示范区上升为国家战略；西递、宏村、古徽州文化旅游区晋级5A级景区；2015年旅游接待量4666万人次，旅游总收入超过400亿元。我市成为国家智慧旅游试点城市、首个国家级旅游服务标准化示范市。黄山风景区荣获全球首个世界优秀目的地景区，黄山风景区管委会作为亚太地区唯一代表参与制订《全球目的地可持续旅游标准》。黄山旅游集团连续7年跻身中国旅游集团20强。国家服务业综合改革试点全面完成，黄山现代服务业产业园获省政府批准设立。全市服务业增加值254.9亿元，年均增长10.1%。

——过去的五年，是我市重大项目快速推进、城乡面貌显著变化的五年。始终坚持把项目工作作为经济工作的主抓手，持续加大有效投入，“十二五”累计实施亿元以上重点项目340个，完成投资1200亿元，是“十一五”时期的2倍。合福高铁、绩黄高速、黄祁高速建成通车，黄山人民期盼已久的月潭水库项目正式开工，黄杭铁路、歙黟一级公路加快建设，高速公路通车里程居全省第3位。高铁黄山北站和歙县北站、高铁客运枢纽、玉屏索道改建、黄山农产品物流园（一期）建成运营。花山大桥、文峰桥建成通车，市图书馆、游泳馆、全民健身中心等一批公用服务设施建成使用，新建和改造提升新安江公园、江心洲广场等一批公园广场，中心城区建成区面积新增8.1平方公里，绿化覆盖率达到46.8%，位居全省第一。扎实开展“三治三增三提升”行动，区县城和建制镇建设步伐加快，黎阳IN巷建成开街，屯光、汤口等8个镇跻身全国重点镇。全市城镇化率由2010年的43.3%提高到51.8%。扎实开展改徽建徽和“三线三边”环境整治，美好乡村建设走在全省前列，徽州区、黄山区、屯溪区荣获安徽省2013年度美好乡村建设先进县称号。

我市荣获全国无障碍建设创建城市、数字城市建设示范市称号，在中部六省率先摘取中国人居环境奖城市桂冠。歙县蝉联三届全国文明县城称号。屯溪区荣获“全国和谐社区建设示范城区”。

——过去的五年，是我市改革开放深入推进的五年。完成新一轮市县政府机构改革，市政府工作部门由36个精减为32个，区县政府工作部门减少到24个以下；理顺了区县市场监管体制。继续深化行政审批制度改革，市县两级权力清单、责任清单和涉企收费清单公布施行，市级行政权力事项和行政审批项目分别精简70.6%、52%，实现90%以上行政审批主体向一个内设机构集中、机构向各级政务服务中心集中。建成全市统一的公共资源交易平台。“两个中心、两个工作站”建设经验在全省推广。稳步实施商事制度改革，全市实有市场主体8.7万户，比“十一五”末增加64.2%。新增中国驰名商标15件。大力推进多层次资本市场建设，全市新三板挂牌企业4户，省股权托管交易中心挂牌企业62户。深化农村综合改革，扎实开展农村土地承包经营权确权登记颁证工作，黟县试点工作全面完成。开展农村集体产权股份制改革试点，屯溪区村集体资产股份制改革工作走在全省前列。积极探索农民资金互助社建设，在全省率先完成农村合作金融机构改制任务。深入推进林权制度改革，江南林业产权交易所挂牌运营，基本建成覆盖全省的综合型农林资源交易平台。成功举办了联合国教科文组织黄山对话会、全球可持续旅游委员会黄山年会、中国—东盟媒体论坛、国际旅行商大会等一系列重大活动。我市与瑞典瓦拉市、德国斯特拉尔松德市缔结友好城市，黄山风景区与加拿大班芙国家公园、柬埔寨吴哥窟景区缔结友好景区。黄山屯溪机场升格为国际机场，黄山航空口岸对外国籍飞机开放，台湾居民口岸签注获批启用，开通境外航线6条。加强与长三角、海西经济区等重点区域对接，主动融入杭州都市圈，强力推进招商引资，4家世界500强、15家国内500强企业落户我市。

——过去的五年，是我市生态、文化优势日益彰显的五年。国家主体功能区建设试点示范、国家生态文明先行示范区和国家级生态保护与建设示范区获批。实施全国首个跨省流域新安江生态补偿机制试点工作，第一轮试点任务全面完成。太平湖成功争创国家湿地公园。歙县跻身国家可持续发展实验区。组织开展千万亩森林增长工程，全市森林覆盖率达82.9%，获国家森林城市称号。祁门县获全国生态示范区称号，森林覆盖率位居全国前列。实施黄山松材线虫病三道防线项目，确保了黄山松的安全。认真落实节能减排工作责任制，全面完成省政府下达的节能减排和大气污染防治目标任务，环境质量稳居全省前列。全面启动徽州古建筑保护利用工程，依法有序推进古民居产权流转改革，“百村千幢”工程入选国家文化创新工程，徽州府衙修复工程获中国建筑工程鲁班奖；新增国保单位14处、国家级历史文化名镇名村名街6处、中国传统村落68个。深入推进徽州生态文化保护实验区建设，成功举办两届中国非物质文化遗产传统技艺大展，新增国家级非物质文化遗产名录4项、国家级非遗传承人9人，新增国家级非遗生产性示范基地1处。建成国家级文化产业基地1个、省级文化产业园区1个、省级文化产业基地9个，文化产业增加值达41.5亿元、年均增长11%。《大黄山》电视纪录片获全国精神文明建设“五个一”工程奖。

——过去的五年，是我市社会事业稳步发展的五年。扎实开展创新型城市建设，建成国家级创新平台3个，国家级高新技术企业达79家、是“十一五”时期的2.1倍，我市荣获“全国科技进步先进市”。全市义务教育学校标准化覆盖率由2010年的不足10%提高到98.7%，新改扩建公办幼儿园135所。实施校舍安全项目202个。黄山区、休宁县、黟县通过国家义务教育发展基本均衡县（区）认定。组建黄山职业技术学院，目前在校生6300余人，安徽省行知学校、黄山旅游管理学校进入国家中职改革发展示范学校行列。市级公立医院改革全力推进，县级公立医院改革初见成效。市县两级公立医院改扩建项目基本完成，新安名医堂开诊。新型农村合作医疗参合率达101.04%。实施单独两孩政策，适度低生育水平保持稳定。大力发展群众体育、竞技体育和体育产业，入选中国十佳运动休闲城市，成为国家体育产业联系点城市，我市著名运动员吕秀芝先后在奥运会、亚运会、田径世锦赛上取得佳绩，张世腾在全国第九届残运会上勇夺金牌。圆满完成第八届、第九届村委会换届选举工作，建成精品社区40个。扎实开展“六五”普法，加大矛盾纠纷排查化解力度，认真办理信访事项，深入开展平安创建，荣获全国社会管理综合治理优秀市“三连冠”，成功捧回“长安杯”。军民融合深度发展迈出了新步伐，圆满完成了国防动员实兵演习，民兵整组、兵员征集、人民防空和双拥优抚安置工作成效明显，荣获“全国双拥模范城”三连冠、“全省双拥模范城”六连冠。全面落实安全生产责任制，5年未发生重特大事故，连续12年获得全省安全生产先进单位。妇女、未成年人、老年人、残疾人权益得到保障。新闻出版、文学艺术、哲学社会科学、统计、档案、地方志等工作得到加强，气象、地震、防灾减灾等工作取得新进展，民族宗教、外事侨务、对台等工作取得新成绩。

——过去的五年，是我市人民生活持续改善的五年。坚持每年将财政新增支出的90%以上投入民生，“十二五”累计安排民生类资金510亿元，为“十一五”的3.3倍。累计滚动实施48项省市民生工程，惠及全市城乡居民，连续三年荣获全省民生工程绩效考评先进市称号。坚持就业优先战略，城镇登记失业率始终控制在4.5%以内。持续扩大社会保障覆盖面，五项社会保险参保人数93万人次，城乡居民社会养老保险参保人数80.6万人。企业离退休人员养老金和城乡低保、农村五保、重点优抚对象补助标准稳步提高并按时足额发放，省内异地就医实现即时结算。大病保险全面启动，城乡医疗救助9.1万人次。新开工建设各类保障性住房和棚户区改造安置房3.6万套，发放廉租住房租赁补贴5452万元，累计解决或改善4.6万户中低收入家庭的住房困难。加大扶贫开发力度，2012～2014年每年实现2万人左右稳定脱贫。扎实做好地质灾害防治工作，1.16万人消除地质灾害隐患威胁。解决了33.7万农村居民饮水安全。全面落实惠农政策，农民人均受益2617元。

——过去的五年，是政府自身建设不断加强的五年。我们扎实开展党的群众路线教育实践活动和“三严三实”专题教育，坚持把纪律和规矩挺在前面，严格执行中央“八项规定”精神和省、市委有关规定，驰而不息纠“四风”、转作风。认真落实“三个清单”制度，切实做到法定职责必须为、法无授权不可为。出台了进一步规范政府系统重大事项决策行为的实施意见，严格按照法定权限和程序行使权力、履行职责。建立完善新闻发布制度，推进政务公开和政府信息公开。深入开展机关作风集中整治和效能、政风、行风“三位一体”考核评议，认真办好“政风行风热线”、“市民连线”、“市民热线”、“市长热线”和“市长信箱”，进一步拓宽民意表达渠道。全力配合市人大开展执法检查、调研视察，支持市政协开展专项调研、专题协商。推动人大代表建议和政协提案办理由“答复满意”向“结果满意”转变。严格遵守《中国共产党廉洁自律准则》和《中国共产党纪律处分条例》，认真落实党风廉政建设责任制，扎实开展廉政风险防控，强化行政监察和审计监督。

各位代表！刚刚过去的2015年，是全面完成“十二五”规划的收官之年，是黄山市改革发展承上启下的关键之年。一年来，我们坚持精准发力稳增长。认真贯彻落实国家和省一系列稳增长政策措施，及时出台了贯彻落实加快调结构转方式促升级行动计划的实施意见、促进重点产业发展“1+12”政策体系等，全市经济社会发展呈现缓中趋稳、稳中向好的态势。初步测算，地区生产总值同比增长6%，财政收入增长2.5%，固定资产投资增长0.8%，规上工业增加值增长3.8%，社会消费品零售总额增长11.1%，旅游接待量增长12%，旅游总收入增长13%。坚持招商引资增后劲。建立完善市级领导推进重点招商项目等制度，组建6支专业小分队在北京、上海、杭州等地积极开展驻地招商，海亮集团生态农业、超威集团动力能源、安徽神剑新材料、浪潮云计算产业基地、中国黄山国际户外运动基地等一大批项目成功签约，中建材黄山新型显示材料基地、“欧洲之星”欢乐谷等项目开工建设，“讯飞爱途”智慧旅游、京东商城黄山馆上线运营。全年新签项目到位资金131亿元、增长9%。坚持改革创新添活力。全面推进黄山旅游集团等市属国有企业改革，黄山城投集团有限公司挂牌成立。深化国有资产管理改革，实行市直单位的国有房产和经营性资产统一管理、市场化运作。推进金融改革创新，省股权托管交易中心文化旅游板（黄山专板）顺利开板，首批50户企业集中挂牌，首只产业投资基金安元现代服务业投资基金和首只市城市发展基金设立，黄山城投集团有限公司9亿元企业债成功发行。积极探索政府与社会资本合作（PPP）融资新模式，黄千高速等5个项目进入国家项目库。黄山口岸落地签证获批，黄山机场免税店投入运营，新认定黄山百大、猴坑茶业、超港食品等8家离境退税商店。稳步实施商事制度改革，推动“大众创业、万众创新”，全市新登记企业3836户、增长54.9%。我市荣获全国“十大创客之城”、“十大最具文化创意旅游城市”。坚持统筹城乡抓建设。黄山经济开发区区划调整顺利完成。加快推进中心城区“一环三片”建设，205国道改建及梅林南路、黄山路人防地下商业街等项目开工兴建，中心城区排污口治理、垃圾处理、节能照明、交通拥堵节点改造等项目加快实施。加快区县城和建制镇建设，我市荣获新型城镇化建设“中国示范城市”称号。扎实推进美好乡村建设，2014年度55个省级中心村建设基本完成，2015年51个省级中心村建设扎实推进，歙县雄村镇、黄山区庄里村和黟县卢村入选全国特色景观旅游名镇名村。坚持绿色发展强优势。在全省率先全面启动农药集中配送，新安江流域生态补偿机制试点首次写入国家《生态文明体制改革总体方案》，入选2015年中国改革十大年度案例，新一轮试点启动实施。太平湖国家生态环境保护专项稳步推进。坚持以人为本惠民生。实施屯溪一中、屯溪四中和市实验小学联动搬迁，优化中心城区教育布局。统筹做好社保、就业、文化、卫生、体育和食品药品等各项工作。加大精准扶贫力度，1.8万人稳定脱贫。扎实开展安全生产“铸安”行动，加大矛盾纠纷排查和信访积案化解力度，依法严厉打击各类违法犯罪行为，全市社会大局保持和谐稳定。我市被中央文明委授予“全国文明城市提名城市”称号。

各位代表，成绩来之不易，成就令人振奋。这是省委、省政府和市委坚强领导的结果，是市人大及其常委会和市政协监督支持的结果，是全市人民凝心聚力、真抓实干的结果，是历届班子开拓进取、久久为功的结果。在此，我代表市人民政府，向全市广大工人、农民、知识分子、干部，向驻黄解放军指战员、武警官兵和政法干警，向各民主党派、各人民团体和各界人士，致以崇高的敬意！向关心、支持黄山现代化建设的中央、省驻黄单位和海内外友好人士，向在我市创业的投资者、建设者，表示衷心的感谢！

在肯定成绩的同时，我们必须清醒地看到经济社会发展中还存在不少困难和问题：实体经济经营困难、产业支撑不足、财源增长乏力，经济持续增长的基础不稳固，转型发展的任务十分繁重；深化行政审批制度改革、理顺城市管理体制等工作涉及深层次问题和较大利益调整，还需下更大气力；城乡公共基础设施建设欠账较多，就业、教育、卫生、社会保障等方面还存在不少薄弱环节；部分城市低收入群体和深山区、库区、老区群众生活依然困难，精准脱贫的任务相当艰巨。各级政府工作人员运用法治思维、法治方式解决问题的能力和水平也还需进一步提高，政府机关作风和效能建设有待进一步加强。对此，我们一定要高度重视，采取切实有效措施，认真加以解决，不辜负全市人民和各位代表的期望和重托。

二、“十三五”时期指导思想和奋斗目标

“十三五”时期是全面建成小康社会的决胜阶段。一方面我们要看到经济下行的压力依然较大、改革发展深层次矛盾日益凸显，另一方面更要看到国家推动“一带一路”、长江经济带、生态文明建设、全面脱贫攻坚和省委省政府实施“调转促”行动计划、加快推进皖南国际文化旅游示范区建设给我们带来的叠加机遇。综合判断，挑战与机遇并存，机遇大于挑战，黄山仍处于大有可为的重要战略机遇期。我们要主动适应引领新常态，以政府有作为、重担当的良好形象，凝聚起实干兴市的强大合力，奋力开创全市改革发展新局面。

根据《中共黄山市委关于制定国民经济和社会发展第十三个五年规划的建议》（以下简称《建议》），我市“十三五”时期发展的指导思想是：全面贯彻党的十八大和十八届三中、四中、五中全会精神，以马克思列宁主义、毛泽东思想、邓小平理论、“三个代表”重要思想、科学发展观为指导，深入贯彻习近平总书记系列重要讲话精神，认真落实“四个全面”战略布局，坚持创新发展、协调发展、绿色发展、开放发展、共享发展，以提高发展质量和效益为中心，以加快调结构转方式促升级为主抓手，以增进人民福祉、促进人的全面发展为出发点和落脚点，加快推进新型工业化进程，积极推进“旅游+”，着力形成引领经济发展新常态的体制机制和发展方式，加快建设美丽中国先行区，把黄山打造得更美丽、更富裕、更文明。

奋斗目标是：力争到“十三五”末，人均主要经济指标走在全省前列、人均主要社会生态指标走在全国前列，全面建成惠及全市人民的小康社会。

在市委《建议》的指导下，市政府编制了《黄山市国民经济和社会发展第十三个五年规划纲要（草案）》，已提交各位代表，一并请予审议。

各位代表，路虽远，行则必至；事虽难，做则必成！我们坚信五年后的黄山：

——综合实力更强。深入推进大众创业、万众创新，进一步创造新供给、培育新动力，努力保持经济中高速增长，提前实现地区生产总值比2010年翻一番，力争到2020年，全市生产总值年均增长8%左右、达到850亿元并向千亿元冲刺，财政收入突破130亿元，累计完成全社会固定资产投资3000亿元以上，社会消费品零售总额达到410亿元，努力实现新旧发展动能的加快转换，把全市发展提高到一个新水平。

——产业结构更优。以“调转促”行动计划为抓手，补齐短板、做足优势，力争到2020年，全市三次产业结构比重为7∶43∶50，工业增加值突破300亿元，园区工业产值占全市工业产值比重超过90%；国家高新技术企业超100家，高新技术产业增加值占规上工业企业增加值比重达到40%；建成高效生态茶园35万亩，发展家庭农场2000家，培育亿元以上农业产业化龙头企业40户，农业增加值年均增长3.5%；全市旅游接待量突破6500万人次，旅游总收入突破650亿元；服务业增加值420亿元以上，占全市生产总值比重超过50%，努力实现产业发展保持中高速、产业水平迈向中高端。

——生态环境更佳。牢固树立“绿水青山就是金山银山”的发展理念，积极构建符合生态文明建设要求的空间格局、产业结构、生产方式和生活方式，不越生态“红线”，守住发展“绿线”，确保单位地区生产总值能耗、主要污染物排放控制在省下达指标内，森林覆盖率、水环境、空气质量和土壤环境质量继续保持全国领先水平，新一轮新安江流域生态补偿机制试点成效显著，国家生态文明先行示范区建设任务基本完成，让黄山的天更蓝、地更净、水更清、山更绿。

——城市乡村更美。以新型城镇化为引领，将城市和乡村作为一个有机系统进行统筹谋划，促进城乡在规划布局、基础设施、公共服务、生态保护等方面相互融合、共同发展，强化中心城区和区县城辐射带动作用，加快建设一批特色小镇，力争到2020年，全市所有布点中心村和城市社区达到美丽乡村、美丽社区建设要求，努力将我市建成安徽区域性中心城市，打造城乡居民安居乐业的幸福家园。

——文化特色更浓。抓住皖南国际文化旅游示范区和徽州文化生态保护实验区建设的契机，继续实施徽州古建筑保护利用工程，着力培育一批文化领军企业、打造一批知名文化品牌、引进一批新的文化业态、建设一批高水平的文化产业园区和产业基地，在更高层面实现徽州文化保护利用与经济社会发展的互惠共赢，努力打造中国优秀传统文化传承创新区，让古老的徽州文化焕发新的活力、迸发新的光彩。

——人民生活更好。坚持富民与安民共同推进，大力推进就业惠民和创业富民，坚决打赢脱贫攻坚战，建立更加公平更可持续的社会保障制度，加强和创新社会治理方式，到2020年，城镇、农村常住居民人均可支配收入均比2010年翻一番，贫困人口提前全部脱贫，就业、社保、教育、医疗、文化、住房等公共服务体系更加健全，基本公共服务水平进一步提高，让全市人民共建共享幸福美好新生活。

——文明程度更高。坚持两手抓、两手都要硬，把精神文明建设贯穿改革开放和现代化建设全过程，大力弘扬社会主义核心价值观，加快构建社会信用体系，着力提升社会文明程度，努力打造全国学习中华传统文化、践行社会主义核心价值观的重要教育基地，塑造“黄山好人”品牌，成功争创全国文明城市，让向上向善、诚信互助、创新创业的时代精神在黄山蔚然成风，让“梦幻黄山·礼仪徽州”的城市品牌享誉海内外。

三、2016年主要工作

今年是全面建成小康社会决胜阶段的开局之年，也是全面实施“调转促”行动计划的攻坚之年。我们要牢固树立和贯彻落实创新、协调、绿色、开放、共享的发展理念，坚持改革开放，坚持稳中求进工作总基调，按照市委的决策部署，大力发展新型工业和“旅游+”，积极培育新经济新业态，统筹推进城乡发展，着力保障和改善民生，加强和创新社会治理，促进经济平稳健康发展和社会和谐稳定。

今年经济社会发展的主要预期目标是：全市地区生产总值增长8%左右，财政收入增长7%，全社会固定资产投资增长8%，社会消费品零售总额增长10%，城镇常住居民人均可支配收入增长9%，农村常住居民人均可支配收入增长10%，居民消费价格涨幅控制在3%以内，万元生产总值能耗、主要污染物排放总量控制在省政府下达目标以内。在实际工作中，力求发展得更快更好一些。

实现上述目标任务，必须着力做好以下八个方面工作：

（一）着力扩大有效投入。全力抓好重点项目建设，创新招商引资工作方式，切实发挥有效投资对稳增长调结构促升级的关键作用。

强力推进重点项目建设。按照经济工作项目化、项目工作责任化、责任工作考核化和“四督四保”要求，落实市级领导联系、分级调度、督查稽查等项目工作制度，全力以赴抓好332个市级重点项目建设，确保全年亿元以上项目开工97个，竣工36个，完成投资278亿元。全力提速月潭水库、黄杭铁路、歙黟一级公路、宁国至黄山天然气支线工程、黄山电器新型功率半导体器件、昌辉产业园、中建材黄山新型显示材料、超威动力能源、东晶光电二期扩建、美丽之冠国际旅游城等在建项目，加快推进黄千、济祁、黄芜高速前期工作，开工建设神剑新材料、绿色包装产业集聚发展基地、浪潮云计算产业基地、五维科技园、黄山智谷科技园、国药祁红、苏宁生活广场、红星美凯龙家具广场等项目，力争金马股份新增80万套汽车线束生产线、智能手机芯片和整机生产基地、天香科技、齐云山旅游集散中心、奇瑞黄山宿营地等项目建成运营。

持续推进招大引强。加强与长三角、京津冀、环渤海和海西经济区等重点区域对接与交流，持续深化与杭州多层面合作，加速融入杭州都市圈。持续推进精准招商、专业招商、以商引商和小分队招商，瞄准央企、外企和知名民企，着力引进一批带动力强、产业链长的重大项目，力争联合利华茶叶深加工、光大国际战略合作、荣盛集团综合开发、粤海集团城市公用基础设施建设与运营合作、新华发行集团花山谜窟—渐江风景区综合保护与利用等一批重点在谈项目早日签约落地。全年新签亿元以上项目120个，实际到位资金150亿元。

切实提升项目工作水平。抓住国家适度扩大总需求、着力加强供给侧结构性改革的机遇，依托皖南国际文化旅游示范区等战略平台，对接“中国制造2025”，紧盯省调转促“4105”行动计划，加强政策研究，精心谋划编制高水平、有深度、牵动力强的重大项目，争取更多的项目进入国家、省“十三五”相关专业规划和三年滚动投资计划。加强项目储备，形成梯次接续、滚动实施的良性格局。完善重大项目谋划、前期工作、建设、要素保障等推进机制，明确各地、各有关部门的职责，全面提升项目服务水平。

（二）着力推进产业转型。聚焦产业发展，壮大实体经济，着力做精一产、做强二产、做优三产。

强力推进工业突破。全面落实国家和省一系列稳增长政策措施，组织实施“战略性新兴产业集聚发展、传统产业改造提升、民营经济提升、园区转型升级”等四项重点工程，力争全年规上工业增加值增长10%。加大对40户左右重点骨干企业的扶持力度，着力打造黄山工业发展“市级队”。实施“个转企”、“小升规”成长计划，力争全年净增规上工业企业30户以上。全力支持中小企业实施技术改造和科技创新，力争新认定国家高新技术企业6户，省级“专精特新”企业10户以上，全市高新技术产业增加值45亿元。大力培育各类创业创新主体，积极发展众创、众包、众扶、众筹新业态新模式，在全市各类园区培育一批小微企业创业创新基地和创客空间、创新工场。积极申报省现代服务业、绿色包装等战略性新兴产业集聚发展基地，探索推进市战略性新兴产业集聚发展基地建设，力争战略性新兴产业产值增长12%。推动全市各类工业园区转型发展、错位发展，培育壮大市经济开发区绿色包装、电子信息、智能制造等三大主导产业，进一步夯实市经济开发区产业发展基础，支持歙县、徽州区联手打造国家级低碳高新开发区，力争全市园区固定资产投资、工业总产值均增长10%以上。

大力发展全域旅游。按照“全域化规划、全景区建设、全行业融合”的总体要求，落实“一个核心，五个一批”的战略部署，高水平推进皖南国际文化旅游示范区建设，积极推进“旅游＋”行动计划，全面打造幸福旅游、美丽旅游、智慧旅游、信用旅游，力争全年游客接待量增长11%，旅游总收入增长12%，入境游客增长10%，旅游创汇增长11%。强化黄山风景区综合管理，发挥黄山旅游集团全域旅游资源开发的投融资平台作用，启动黄山风景区东海景区规划编制工作，实施山上山下一体化综合开发，大力推进东黄山建设，力争年内在发展定位、基础设施建设、招商引资等方面取得实质性工作进展。启动太平湖、牯牛降5A级景区创建工作，确保齐云山景区通过国家旅游局组织的景观价值评定。深入实施乡村旅游“百千工程”和富民工程，加快建设一批特色旅游小镇（村）、名镇名村，大力开发民宿旅游，推出一批“徽州民宿”，大力发展摄影写生、研修旅行、宗族寻根、健康养生等旅游新业态，进一步打响“中国摄影之乡”品牌。加快推进黄山168国际徒步探险基地、中国黄山国际户外运动基地等重大体育旅游项目建设，持续打造黄山（黟县）国际山地车节、黄山国际登山大会、齐云山万人养生国际徒步大会等国内外有影响力的赛事品牌。加大“梦幻黄山·礼仪徽州”城市品牌营销力度，抢抓杭州G20峰会、上海迪士尼开园机遇，加强游客互送、推进深度合作。办好中国黄山旅游节。积极发挥旅游行业协会和“中国山岳旅游联盟”的作用，主动融入国内外知名旅游企业的营销链。进一步扩大口岸开放，争取开通马来西亚、泰国包机航线，积极拓展东盟市场。鼓励市内景区、景点并购联合，支持旅游企业与资本市场对接，加快培育一批更有经营实力的领军企业。扎实抓好智慧旅游试点，支持“讯飞爱途”打造成全国知名的旅游电商平台。加强旅游应急救援力量建设，开展旅游市场秩序常态化治理，努力营造安全优质舒适的旅游环境。

加快发展现代农业。积极推进全省现代生态农业产业化示范市建设，组织实施绿色增效、品牌拓展、主体培育、科技支撑、产业融合、改革创新“六大行动”，加快转变农业发展方式，全年农业增加值增长3.5%以上。坚持以工业化的理念发展现代农业，着力提升徽茶产业品牌化、规模化、清洁化发展水平，推动毛竹、油茶、枇杷、菊花、山核桃、香榧、食用菌和皖南花猪、泉水鱼等特色种养业规模化经营。争取“太平猴魁茶”国家地理标志产品保护示范区通过国家验收。加快以茶产业为主的农产品精深加工“甲级队”建设，新增亿元以上龙头企业2～3家，全市规模以上农产品加工产值突破200亿元，其中茶产业加工产值65亿元。加快培育新型农业经营主体，引导农民专业合作社成立联合社，新发展家庭农场150个，培育各类现代农业产业化联合体15个。支持龙头企业开展物联网技术示范应用，加快农产品电子商务平台建设，全年电子商务交易额突破6亿元。建立健全农产品质量安全追溯机制，强化重大动植物疫病防控，确保农产品质量安全。

积极发展现代服务业。深化黄山现代服务业产业园控制性详细规划和专项规划，完善园区基础设施，提高园区承载力。加速推进向上创业小镇、金融小镇等重点项目建设，开工建设智慧小镇、黄山声谷产业园、智慧交通黄山基地，再谋划和引进一批项目，着力打造国家旅游信息服务业集聚基地和全市新的经济增长极。实施屯溪老街、花溪饭店改造提升，打造屯溪老街—黎阳IN巷—外边溪城市核心商圈。抓好皖新现代物流园、黄山农产品物流园、徽州现代物流园等重大项目建设，全面提升省级服务业集聚区功能。推动大数据和云计算运用，大力发展文化创意、健康管理、动漫影视等新兴产业，力争服务业增加值增长8%，规上文化企业超40家，文化产业增加值增长15%以上。落实中央、省房地产去库存政策，建立购租并举的住房制度，化解问题楼盘，促进房地产市场健康发展。

（三）着力统筹城乡发展。坚持以新型城镇化为引领，加快构建中心城区与各组团、区县城、重点集镇、中心村协调发展新格局。

加强城市规划管理。积极探索“多规合一”，加快编制市域空间管控规划，有效协调各类专项规划，全面提升规划的前瞻性、科学性和严肃性。划定全市城市发展空间、重大基础设施建设廊道空间，强化空间资源配置管控，统筹地下地上工作，完成中心城区地下空间综合利用规划编制。加强中心城区和区县城重要节点的城市设计，彰显文化特色，提升建设品质。深化城市管理体制改革创新，全面推进美丽社区建设，加快打造“智慧城市”和数字化城管平台，实现中心城区社区公共服务信息平台全覆盖，争取用2～3年时间实现城市管理精细化、网格化、信息化，进一步提高城市管理水平。从细节入手，深化全国文明城市创建工作，积极争创联合国人居奖城市、国家卫生城市、国家环保模范城市和国家生态园林城市，实施“五城联创”。

提高城市建设水平。坚决打赢征迁安置和控违拆违攻坚战，全面提速205国道改造、梅林南路等项目建设，加快发展市经济开发区新城组团，启动花山片区开发，完善江南片区基础设施和服务设施，构筑中心城区“一环三片”。完成黄山路及百大交口交通整治，贯通新园西路，逐步打通中心城区交通微循环。实施滨江中路等人行道及绿化提升改造，启动公共停车场、自行车租赁系统建设。申报国家海绵城市试点，建成地下管线信息系统。编制实施中心城区棚户区改造和老旧小区整治三年计划，2016年改造棚户区6个、整治老旧小区85个。积极运用政府与社会资本合作（PPP）模式，引导社会资本投资、参与城市建设和运营。积极推进区县城“三治三增三提升”行动。推动城市基础设施互联互通、共建共享，加快屯休、屯徽、歙徽同城化建设步伐。扶持10个左右中心镇，打造产业特色鲜明、人文气息浓厚、生态环境优美、功能叠加融合、体制机制健全的特色小镇。有序推进农村转移人口市民化，力争全市户籍人口城镇化率提高2个百分点。

推进美丽乡村建设。按照“以点为主”向“由点到面”战略转换的要求，扎实推进“三线四环三片”特色示范区建设，积极做好黄山区整县推进试点工作。在全面完成2014年度55个省级中心村建设任务的同时，重点抓好2015年度51个省级中心村建设，全面启动2016年度49个乡镇政府驻地建成区整治建设和43个省级中心村建设。实施农村公路畅通工程，加强以“八小”水利改造提升工程为主的农田水利基本建设。突出市场主体，创新投入机制，鼓励和引导社会力量参与美丽乡村建设。统筹推进中心村产业发展、农村环境治理、“三线三边”环境整治以及农村社区建设和文明创建，逐步壮大村集体经济实力，确保美丽乡村建设走在全省前列。

（四）着力强化财政金融支撑。进一步优化财政收支结构，强化金融支持保障，切实增强服务经济发展的能力。

提高财政运行质量。把培植税源作为组织收入的着力点，多措并举强化财源建设。着力抓好“1+12”产业扶持政策落实，进一步引入竞争性分配机制，提升财政资金支持重点产业发展效果。用活营改增过渡期政策，加大中小企业注资、担保、股权投资和贴息力度。加大中央和省均衡性转移支付、专项转移支付补助的争取力度，进一步盘活财政存量资金，加大资金统筹使用力度，做好市直单位国有房产和经营性资产统筹管理、划转移交等后续工作。严格支出管理，规范预算执行，大力压缩“三公”经费，一般公用经费实行“零增长”。将政府债务纳入预算管理，做好存量债务置换工作，进一步做实政府融资平台，发挥政府债券在筹措建设资金、降低融资成本、防控债务风险等方面的积极作用。

提升金融服务水平。加大金融支持服务实体经济力度，落实企业应急还贷机制，加快推广“税融通”、“4321”政银担银企风险分担合作机制、小额贷款保证保险等模式，力争全年贷款增幅达到全省平均水平。加快多层次资本市场体系建设，充分发挥安元现代服务业产业投资基金作用，积极推动省文化旅游投资基金落户注册我市，支持发展天使投资基金、创业投资基金和股权投资基金等。用好省股权托管交易中心文化旅游板（黄山专板），全年新增首发上市企业1家，报省辅导备案企业4家，新三板挂牌企业5家以上，力争黄山城投集团公司发行短期融资券、公司债和融资租赁取得突破，黄山旅游股份公司资本市场融资取得新进展。稳步推进村镇银行县域全覆盖工作，实现行政村基础金融服务“村村通”。推进农村住房财产抵押贷款试点和黄山区全国土地承包经营权抵押贷款试点，推动市及区县供销农副产品专业合作社开展信用合作。探索政银担合作共建中小企业统借统贷平台和风险补偿机制，完成市信用担保集团公司“1+6”集团模式搭建。严守风险底线，严厉打击各类非法集资行为，确保全市金融秩序稳定。

（五）着力厚植特色优势。坚持特色、强化优势，使望得见山、看得见水、记得住乡愁成为黄山的鲜明标识。

加大生态环境保护力度。加快国家生态文明先行示范区、国家生态保护与建设示范区、国家主体功能区试点示范建设，力争黄山区、歙县等五个区县获批国家重点生态功能区。以实施新一轮新安江流域生态补偿机制试点为重点，系统推进新安江流域水污染防治、水生态保护和水资源管理，探索设立新安江生态保护和产业发展基金，力争全年完成投资16.5亿元。加快太平湖生态环境保护专项建设，高标准建设国家湿地公园。新造林3.55万亩，全面完成千万亩森林增长工程。加大生物多样性保护，抓好松材线虫病防控三期项目建设。加强水土保持工作。完成30处以上地质灾害隐患点治理任务。深化国家和省级生态县、生态乡镇和生态村创建，落实生态环境损害责任终身追究和赔偿制度。

强化资源节约集约利用。大力推广高效无残留和生物农药，力争两年内基本建成农药集中配送体系，并逐步将化肥列入集中配送网络。实施大气污染和水污染目标责任管理，基本淘汰黄标车，确保大气环境和地表水质量继续在全省保持领先。开工建设城市生活垃圾焚烧项目，建成日处理150吨污泥、餐厨废弃物综合处理和市建筑垃圾处置场项目。完善城乡污水收集和垃圾收集转运系统。深入推进生态、循环经济示范创建，开展重点用能行业对标达标活动。立足长远、通盘考虑，科学划定永久基本农田，认真做好土地利用总体规划调整完善工作，确保全市耕地保有量和基本农田保护面积不减少，力争土地卫片执法检查“零约谈、零问责”。扎实开展批而未供和闲置土地处置攻坚行动，提高土地资源利用效率。

推进文化传承创新。深入实施徽州古建筑保护利用工程，着力推进120个、年投资6亿元的保护利用项目。深化古民居流转改革，加快建立产权流转交易平台，建立健全保护利用的新机制新模式。加强与联合国教科文组织亚太世界遗产中心合作，启动皖南古村落世界遗产拓展项目、歙县古徽州城墙申遗和“千年古县”申报工作。完成全市可移动文物普查，力争黟县获批国家历史文化名城。推进徽州文化生态保护实验区建设，建设一批国家非遗传习中心。开展徽墨歙砚、万安罗盘、徽州漆器、徽州四雕等生产性保护，新增国家级非遗传承人10人以上。出版发行《徽州文化大辞典》，启动国家级名镇名村名街志编纂工作。积极开展公共文化服务标准化、均等化试点工作，推动公共文化服务向社区和农村基层延伸。创作生产一批优秀文化产品，力争2～3个项目入围国家艺术基金资助项目。抓好文化市场监督管理，推动文化市场繁荣有序。

（六）着力深化重点改革。坚持问题导向，强化责任担当，把改革落准落细落实，使改革更加精准地对接发展所需、基层所盼、民心所向，更好造福群众。

加快转变政府职能。深化行政审批制度改革，继续做好国务院、省政府取消和下放行政审批事项的衔接、落实工作；建立行政审批中介服务清单，推进政府公共服务清单建设；强化政府权力清单、责任清单和涉企收费清单动态管理，推进政府权力清单网上运行，推广互联网+政务服务。加大财税体制改革，明晰市与屯溪区、市经济开发区、现代服务业产业园事权和支出权责任界定。完善城市管理相对集中行政处罚权工作，推进综合执法。深化公安行政管理改革，全面推进基础信息化、警务实战化、执法规范化、队伍正规化建设。实施市供销体制改革。推进事业单位分类改革。继续深化商事制度改革，全面推行“三证合一、一证一码”，探索企业注册全程电子化。

积极推进改革试点。启动大黄山国家公园创建工作，加快国家生态文明先行示范区建设。制定国家级旅游业改革创新先行区实施方案，积极探索旅游管理相对集中行政处罚权试点，在黟县、黄山区全域和黄山风景区等市内5A级以上景区，以及屯溪老街开展综合执法试点工作。实行放开户口迁移政策，建立城乡统一的户口登记制度，加快推进新型城镇化试点。扎实推进省级市县一体化公共资源交易平台建设试点工作，积极开展碳排放权、排污权、水权等自然资源实行统一平台有偿出让制度。积极推进国有林场改革，深化农村集体产权制度改革和林权制度改革。进一步做大做强江南林权交易所。

加快推进市属国企改革。尊重市场规律和企业发展规律，继续抓好市城投改革转型后集团化运行工作，将其打造成服务城市建设、融资能力更强、经营范围更广的地方骨干企业集团。积极推动黄山旅游集团、市信用担保集团、市文投公司等市属国有企业改革，建立健全现代企业制度，完善法人治理结构。

（七）着力保障和改善民生。坚持以民为本、以人为本，让人民群众有更多的获得感。

扎实开展脱贫攻坚。按照“五个一批”基本路径和“六个精准”的工作要求，把贫困发生率超全市平均水平的47个乡镇和153个贫困村作为脱贫攻坚的重点，把建档立卡贫困人口作为脱贫攻坚的主攻对象，完善单位包村、干部包户、驻村帮扶的工作机制，深入开展“万名党员干部帮万户”行动。全力实施“脱贫攻坚十大工程”，抓好4412人贫困人口易地扶贫搬迁。改善贫困地区生产生活条件，提高贫困人口致富能力，确保全年脱贫人口2万人左右，68个贫困村基本达到出列标准。

巩固提升民生工程。坚持普惠性、保基本、均等化、可持续方向，认真组织实施好省33项民生工程，其中新增农村道路畅通、农产品食品安全、城市老旧小区整治等6项，提高义务教育经费保障机制、新农合、城镇居民医保等6项补助标准。坚持积极而为、量力而行，继续实施市级“菜篮子”民生工程。加强资金管理和质量监管，完善分类管养，强化绩效评估，确保全市民生工程工作继续走在全省前列。

完善就业和保障体系。实施更加积极的就业政策，建成市人力资源市场，鼓励以创业带就业，创造更多就业岗位。大力开展就业援助、就业帮扶和技能培训，统筹推进各类重点群体就业，确保全年城镇新增就业1.8万人，城镇登记失业率控制在4.5%以内。稳步做好机关事业单位养老保险制度改革和城乡养老保险制度衔接，启动城乡统一的医疗保险制度改革，强化五项保险数据比对，提升数据质量，五项保险参保人数92万人次。进一步提高新农合受益面和保障水平，实现政策范围内住院费用报销比例达到70%以上。加快推进公立医院住院患者按病种付费，完善重特大疾病医疗救助政策，推动医疗救助与基本医疗保险、大病保险有效衔接。逐步提高农村“五保户”供养标准。加快公办养老机构改革，支持社会力量兴办养老机构，积极推进医养结合养老模式，促进养老服务从基本生活照料向医疗健康、精神慰藉等方面延伸，提高老年人生活品质。以非公经济单位为重点，进一步扩大住房公积金制度覆盖面。推进棚改安置货币化和住房保障货币化，稳步扩大公租房保障范围。

加快发展社会事业。继续实施第二期学前教育三年行动计划，全市乡镇公办中心幼儿园覆盖率达90%。稳步推进中小学教师“无校籍管理”改革，促进全市义务教育均衡发展。进一步优化教育布局调整，新组建1所一定规模的市属中等职业学校。鼓励黄山职业技术学院与企业开展联合办学。支持黄山学院建设有特色、高水平的应用型大学。深入推进医药卫生体制改革，健全农村医疗卫生服务网络，进一步打响新安医药特色品牌。广泛开展全民健身运动，积极做好十四届省运会备战工作。全面实施一对夫妇生育两孩政策，扎实做好人口和计生工作，深入开展爱国卫生运动，抓好重大疾病防控。开展第三次全国农业普查。积极适应国防和军队改革新形势，围绕探索构建具有黄山特色军民融合深度发展新格局，巩固和深化双拥成果，加强国防教育、国防动员、后备力量建设和人民防空工作，以实际行动支持和服务军队改革。切实维护妇女、儿童和老年人、残疾人合法权益。做好民族、宗教、审计、物价、档案等工作。充分发挥工会、共青团、妇联等人民团体的重要作用。

切实维护社会和谐稳定。全面加强和创新社会治理方式，建设更高水平的“平安黄山”。加快推进社会服务管理信息化平台建设，确保“12345”统一呼叫中心运营。推进黟县农村社区建设试点县建设，力争全市农村社区建设覆盖率达55%。加强人民调解、行政调解、司法调解联动的衔接配合机制，做好社区矫正监管和安置帮教工作。启动实施“七五”普法，加强和规范基层法律服务。扎实开展安全生产“铸安”行动，力保各项安全生产指标控制在省下达范围之内。切实做好森林防火和气象、地震等防灾减灾工作。加大食品药品安全监管力度，创建省级食品药品安全城市。健全社会稳定风险评估机制和重大不稳定问题清单制度，坚持和完善领导干部接访下访制度，认真落实诉访分离规定，加大矛盾纠纷排查、初信初访办理和信访积案化解力度。开展县级矛盾纠纷联调中心建设试点，全面提升“两个中心、两个工作站”规范化建设。加强互联网安全管理，提升网络突发事件处置能力。完善立体化、数字化社会治安防控体系，严密防范、依法打击违法犯罪活动。

（八）着力加强政府自身建设。始终在思想上、政治上、行动上与以习近平同志为总书记的党中央保持高度一致，以忠诚、干净、担当为座右铭，以“三严三实”为行为准则，坚决贯彻落实中央和省、市委的决策部署，奋力争当“四个自觉”模范。

一是建设效能政府。以敬民之心行简政之道，实施政府提速工程，深入开展效能建设，努力为群众和企业提供更加便捷、周到的政务服务。创新体制机制，探索建立行政服务效能综合评价考核机制，健全完善以质量和效益为导向的政府目标管理考评体系，着力构建效能持续提升的长效机制，把“政府提速”传导到每个人，把“马上就办”落实到各环节，坚决整肃庸政懒政怠政。砥砺担当精神，强化宗旨意识，加强学习思考和实践历练，不断研究新情况、解决新问题，摆脱传统增长的路径依赖，提高引领新常态、推动新发展的能力和水平。

二是建设法治政府。认真贯彻落实中共中央、国务院《法治政府建设实施纲要（2015-2020年）》，自觉尊法学法守法用法，建设职能科学、权责法定、执法严明、公开公正、廉洁高效、守法诚信的法治政府。健全依法决策机制，强化决策法定程序的刚性约束，制定出台政府重大行政决策合法性审查程序规定、责任倒查和终身追究办法，推进行政决策科学化、民主化、法治化。加强行政复议工作，提高行政复议公信力。强化权力监督制约，坚持重大决策出台前向人大及其常委会报告、与政协民主协商，广泛听取各民主党派、工商联和无党派人士意见，自觉接受人大及其常委会的法律监督、工作监督和政协的民主监督，认真做好人大代表议案、建议和政协委员提案办理工作。积极推进决策公开、执行公开、管理公开、服务公开、结果公开，主动接受社会公众和新闻舆论监督，确保权力在阳光下运行。

三是建设廉洁政府。严格落实党风廉政建设责任制，着力完善廉政风险防控、监控、处置长效机制，全面推进惩治和预防腐败体系建设。认真贯彻《中国共产党廉洁自律准则》和《中国共产党纪律处分条例》，严格执行中央“八项规定”精神和省、市委有关要求，持之以恒纠正“四风”。把权力关进制度的笼子，严格落实项目招投标、政府采购、国有土地出让等各项制度规定。强化行政监察，建立健全“三单二书一平台”的权力运行和监督机制；用好审计利器，对公共资金、国有资产、国有资源和领导干部履行经济责任情况实行审计全覆盖。始终保持反腐败高压态势，以锲而不舍、驰而不息的决心，不断提高拒腐防变和抵御风险能力，努力实现干部清正、政府清廉、政治清明。

各位代表！百舸争流，奋楫者先；千帆竞渡，勇进者胜。让我们紧密团结在以习近平同志为总书记的党中央周围，在省委、省政府和市委的坚强领导下，励精图治，奋发有为，锐意进取，开拓创新，为加快建设美丽中国先行区、共筑更美更富黄山梦，全面建成小康社会而努力奋斗！
